# Welfare effects of health insurance in Mexico: The case of Seguro Popular de Salud

**DOI:** 10.1371/journal.pone.0199876

**Published:** 2018-07-02

**Authors:** Rocío García-Díaz, Sandra G. Sosa-Rubí, Edson Serván-Mori, Gustavo Nigenda

**Affiliations:** 1 Department of Economics, Monterrey Institute of Technology and Higher Education, Nuevo León, México; 2 Center for Health Systems Research, National Institute of Public Health, Morelos, México; 3 National School of Nursing and Obstetrics, National Autonomous University of México, México City, México; Seoul National University College of Medicine, REPUBLIC OF KOREA

## Abstract

This study contributes with original empirical evidence on the distributional and welfare effects of one of the most important health policies implemented by the Mexican government in the last decade, the *Seguro Popular de Salud* (SPS). We analyze the effect of SPS on households’ welfare using a decomposable index that considers insured and uninsured households’ response to out-of-pocket (OOP) payments using both social welfare weights and inequality aversion. The disaggregation of the welfare index allows us to explore the heterogeneity of the SPS impact on households’ welfare. We applied propensity score matching to reduce the self-selection bias of being SPS insured. Overall results suggest non-conclusive results of the impact of SPS on households’ welfare. When we disaggregated the welfare index by different sub-population groups, our results suggest that households’ beneficiaries of SPS with older adults or living in larger cities are better protected against OOP health care payments than their uninsured counterparts. However, no effect was found among SPS-insured households living in rural and smaller cities, which is a result that could be attributed to limited access to health resources in these regions. Scaling up health insurance coverage is a necessary but not sufficient condition to ensure the protection of SPS coverage against financial risks among the poor.

## Introduction

One of the most important concerns in developing countries is the increasing out-of-pocket (OOP) health care payment made by households and individuals. OOP health payments are the amount of money paid by households to purchase private health services and medicines when members of a household have a health care need. Such health shocks are hardly predictable and among the most important factors associated with the reduction of the welfare of households. The situation is especially severe in developing countries where health protection systems and insurance schemes do not cover the totality of the population, frequently conditioning and limiting access to services for the most disadvantaged [1,2]. Consequently, the provision of health services relies considerably on OOP health payments, which are increasingly recognized as a major consideration in the financing of health care. Considered to be the most inefficient and inequitable means of financing health policy, OOP health payments constitute one of the most important challenges facing health policy [3].

Aiming to increase public expenditure on health care, social, national and public insurance schemes, frequently featuring risk-pooling mechanisms, have been proposed as financing tools to reduce the share of OOP expenditures and in turn the negative impact of these payments on households [1,4–6]. Recently, several developing countries have introduced subsidized or government-run health insurance plans, allowing the poor and workers in the non-formal sector to enroll on a non-contributory basis [7–10]. In 2003, the Mexican government launched a structural reform of its public health sub-system to increase the financial protection of the 50 million people who were not covered by any of the existing social insurance schemes–compulsory contributory insurance covering formal workers-. As part of this reform, *Seguro Popular de Salud* (SPS), a voluntary public health insurance targeting the uninsured poorest groups of the population, was designed and implemented. The government of Mexico established it in an effort to expand health care to the population without health insurance and to reduce health-financing inequities. Since 2004, SPS has gradually expanded to include 55.6 million people. During the Project’s life cycle between 2009 and 2013, 24 million people joined SPS and 22.8 million affiliates received a preventive health risk screening.

According to its design, under this insurance coverage, households make subsidized contributions based on ability to pay to the public fund [11]. Enrollment in SPS is not dependent on health status or pre-existing illness, and there is no co-payment based on the type of health care received. Medical services to the beneficiaries are mainly offered through the public health network, which has increased access to outpatient, hospital and specialized care, in addition to medicines and laboratory services [12,13]. This health insurance is focused on protecting uninsured households from excessive health expenditures that endanger their financial security.

Previous research has shown that SPS has had an overall significant impact on improving access to and utilization of health services and medicines [13–16], reducing health spending and catastrophic health expenditures [11,17–19], and offering financial protection for the poorest groups of the population [20]. However, there is little evidence about the distributional effect of SPS among its recipients, in other words, the magnitude of SPS’ impact on households’ welfare distribution [20]. We analyze the effect of the program on households’ welfare using households’ income distribution after OOP health payments as a measurement of the status of household welfare.

Given the widespread concern about how household welfare might be affected by OOP health payments, this paper focuses on analyzing the SPS welfare effect using an alternative methodology that measures the impact of OOP health payments on households’ income distribution. We follow the distributional characteristic approach, used in marginal welfare analysis [21–24], to provide an alternative measure to analyze the impact of OOP health payments on households’ welfare measured through changes in income distribution after OOP health payments. This distribution of the households’ payment framework considers two aspects: i) the social welfare weights, which result from households’ different responses to changes in household income (this response is different according to the level of household income before OOP health payments and the size of OOP health payments relative to the household income level) and ii) the change in households’ relative position with respect to the income level of other households after OOP health payments.

Although our alternative method complements and shares some similarities with those proposed in the literature [25–28], an important innovation of the proposed methodology is that the index used in this paper is decomposable, thus allowing us to illustrate how the SPS program impacts some policy-relevant subgroups. This might be of crucial importance in order to disentangle the distributional effect of a decentralized policy such as SPS, which entails a group of activities targeted to specific subpopulations or regions of the country. Furthermore, the analysis can be easily extended to verify the robustness of the results by means of the OOP health payments dominance curve analysis presented in Garcia-Diaz and Sosa-Rubi [20]. The OOP health payments dominance curves impose minimal ordinal structure on welfare indices in order to identify non-intersecting income distribution curves and to offer a robust ranking of alternatives.

Based on these elements, our study presents results of the distributional effect of the SPS in Mexico eight years after its initial implementation in 2003. We hypothesized that SPS has positive effects on households’ welfare, a result that could be mediated by the household demographic composition and its geographic location. We use a methodology that considers both changes in levels of household income and shifts in ranking of household income after OOP health expenditures among insured and uninsured poor households. Our methodology also considers the disaggregation of the distributional impact in relevant subgroups. Following recent literature on SPS evaluation, we control for observed heterogeneity through the use of propensity score matching (PSM) to reduce the selection bias attributed to the self-selection of program enrollment [9,29,30].

The paper is organized as follows. Section 2 outlines the materials and methods in the analysis. First, it presents the data and results of the model used to estimate PSM for the analysis of the impact of the program and the balancing test. Then, the distributional welfare impact measure is discussed including its decomposability properties and its extensions to the marginal welfare dominance approach. Section 3 presents the results of the analysis and the final section concludes the paper.

## Materials and methods

### Data, propensity score matching and balancing results

The data for the analysis were requested and obtained from the public survey repository hosted at the National Institute of Statistics and Geography (INEGI). In particular, we use data on household expenditure and health expenditure from the 2010 Mexico Household Income and Expenditure Survey (ENIGH) (see details in http://www3.inegi.org.mx/rnm/index.php/catalog/30). The purpose of this survey was to collect data on the income, spending, sociodemographics, and employment situation of households in Mexico. The data were collected (from August to November 2010) through in-home, in-person interviews. The survey included households in urban and rural areas throughout Mexico.

We estimated the household equivalent expenditure to analyze households’ expenditure distribution. We used a strict definition of SPS affiliation selecting only those households in which all members are covered by the SPS (N = 8,042). This definition is in accordance with the rules of SPS affiliation that covers only those households that do not have any type of social insurance coverage.

In order to have an adequate counterfactual, we selected those households that reported not having any kind of insurance coverage as a comparison group (N = 6,689). Applying the PSM technique [31], we reduce the bias deriving from self-selection to SPS affiliation among observable differences between SPS-insured and uninsured households previously used [13,32,33], such as: age, education, gender, marital status, household asset index, area of residence (north, center and south), place of residence (metropolitan, urban and rural), the report of health needs among households in the last 12 months (the question in the ENIGH refers to any health need: in the last 12 months: Have you been sick, have you had any pain or malaise or have you had an accident which have prevented you from carrying out your daily activities?, and the level of SPS penetration at municipality level among eligible groups of the population (i.e., the program’s coverage level measured through the percentage of the population affiliated among the eligible population).

The matching process was performed using a single nearest-neighborhood algorithm including caliper = 0.001, non-replacement and common support (results of the performance of PSM are shown in S1 Table and S1 Fig). We performed tests to verify balance (see Table 1). We did not find differences in the main characteristics of households who remain in the common support area of the PSM, with the average percentage absolute bias before the matching 26.54% and after matching 1.36%. The interquartile range of the propensity score in the region of common support ranged from 0.047 to 0.927. After the matching process, we ended up with an analytical sample of 11,117 households (SPS = 4,428 and uninsured = 6,689). In this way, insured and uninsured households are more appropriate populations to compare between and, therefore to measure the distributional impact of the program among the target population of the SPS.

**Table 1:** Sample characteristics before and after matching process.

	Full sample		Matched sample	
	With *Seguro Popular de Salud*	Without any health insurance	With *Seguro Popular de Salud*	Without any health insurance
	N = 8,042	N = 6,689	N = 4,428	N = 6,689
	(1)	(2)	(3)	(4)
Head of household (%)				
Male (%)	76.1	69.8**	72.4	72.4
Age (%)				
<20 yrs.	0.4	0.72*	0.5	0.5
20–39 yrs.	36.4	29.2**	33.3	32.5
40–59 yrs.	40.3	36.5**	38.3	39.2
60–79 yrs.	22.9	33.6**	27.9	27.9
Married (%)	76.2	59.9**	68.2	68.0
Schooling (%)				
No Education (0 yrs.)	18.5	12.1**	15.8	15.9
Primary (0–6 yrs)	48.7	38.4**	44.8	45.1
Secondary (6–9 yrs.)	21.6	19.2**	21.7	22.6
High school (≥10 yrs.)	11.2	30.3**	17.7	16.4
Asset Index^φ^	-1.0	-0.11**	-0.6	-0.5
Members with any health problem (%)	26.0	24.9*	25.3	25.9
Residence area (%)				
Rural	42.6	18.8**	28.7	27.3
Urban	34.1	27.0**	33.2	34.6
Metropolitan	23.3	54.1**	38.2	38.1
Geographical region (%)				
North	21.9	21.2	21.2	22.2
Center	32.8	41.9**	37.1	37.2
South	45.3	36.9**	41.6	40.6
Penetration of SPS (%)^Φ^	53.8	37.1**	43.9	43.6

Note

** p<0.01

*p<0.05

+p<0.10. The p-values refer to the differences between households with Seguro Popular de Salud (SPS) and households without any health insurance. Matching process was performed using single nearest neighborhood algorithm including: caliper = 0.001, non-replacement and common support.

^Φ^At municipality level.

^φ^Proxy of household socioeconomic level.

#### The distributional welfare impact of OOP health payments

The analytical approach used in the analysis follows that used by Garcia-Diaz and Sosa-Rubi [20], which explores the distributional poverty impact of OOP health payments using distributional information that assigns different household weights according to the amount of income accrued by households across an income distribution spectrum of the poorest households. The proposed methodology in this paper extends this analysis to measure how changes in income after OOP health care payments, *Δh*, will affect levels of welfare measured through the distributional income and formally defined with a welfare function, *W*. Here we take into account all the household income changes along the whole income distribution, rather than focusing just on the lower part of the distribution (income distribution of the poorest households). In order to do this, we write the social welfare as a function of household prices, *p*, and income, *y*:

$$W(v^{1}(p,y^{1}),\ldots ,v^{i}(p,y^{i}),\ldots ,v^{n}(p,y^{n}))$$ (1)

where v^i^(p,y^i^) is the indirect utility function of household *i = 1*,*…*,*h* whose income is *y*^*i*^ and *p* is the vector of commodity prices faced by that household.

The change in social welfare after the change in household income following the OOP health care payments made by a household *i*, *Δh*^*i*^, is given by the following expression:

$$\Delta W=\int_{0}^{a}\frac{\partial W}{\partial v^{i}(p,y^{i})}\frac{\partial v^{i}(p,y^{i})}{\partial y^{i}}∆h^{i}f(y)\mathrm{dy}=\int_{0}^{a}\beta^{i}∆h^{i}f(y)\mathrm{dy}$$ (2)

This change of welfare shown in the first part of expression (2) can also be specified as the density distribution of household income *f(y)* weighted by the changes in household OOP health payments *Δh*^*i*^ and a social welfare weight, *β*^*i*^. The welfare weight of a household, *i*, *β*^*i*^, is defined as the social valuation of extra income to household *i*. It determines the importance attached to income changes as a result of changes to OOP health care payments made by the household, *Δh*. One commonly used form of utility is the Atkinson’s utility function [34] and its associated welfare weights, giving:

$$\beta^{i}={\left(\frac{1}{y^{i}}\right)}^{\varepsilon }$$ (3)

This function allows the welfare judgments of the policy analyst to be contained in the chosen value of the parameter *ε*. The value of *ε* can be treated as a measure of concern for equity. The extreme case is when ε→∞ gives the function min_*i*_*(y*_*i*_*)*, which only takes into account the effect of OOP health payments by the very lowest income household. At the other extreme, when ε→0 the utility function is a linear function which assigns all welfare weights equal to unity, therefore it implies no concern for equity. This utility function has been used to consider different inequality weights in the measurement of socioeconomic inequalities in health by Wagstaff [35] and Erreygers [36], among other important extensions to the traditional analysis.

The function Δ*W* will differ across distributions because welfare weights will differ across households and because OOP health care payments will also be different across households. The greater the proportion of OOP health care payments paid by the poorest households (i.e., those with a relatively high *β*^*i*^), the higher the value of the distributional welfare impact. The welfare impact and the results can be varied according to the parameter *ε* that captures aversion to inequality, which allows us to incorporate concerns for poverty without the need to introduce a sharp distinction between poor and non-poor household in the analysis, as some methods focused on analyzing the effect of OOP health payments using measures such as catastrophic health expenditure and impoverishment [4,37]. As *ε* increases, we provide greater weight to the poorest households in the income distribution to changes in terms of the OOP health payments they made.

#### Decomposing distributional welfare impacts into subgroups

An important characteristic of (*ΔW*) in Eq (2) is that it is readably decomposable as the sum of contributions of different subgroups in the population. This offers us a consistent breakdown in terms of OOP health payment effects on the income distribution of different population subgroups (considered separately). For that purpose, we let *F*_*j*_*(y)* be the distribution function for the income *y* of households in the *jth* subgroup (where *j* = 1,2,…,*k*), and *θ*_*jF*_ be subgroup’s *j* population share (the number of household in subgroup *j* divided by the total number of households). The social welfare impact in (2) for the population can be re-expressed as the average of subgroup welfare impacts, as follows:

$$\Delta W=\sum \theta_{\mathrm{jF}}\int_{0}^{a}\beta^{i}∆h^{i}f(y)\mathrm{dy}$$ (4)

This allows us a consistent decomposition in terms of OOP health payments effects by subgroup components (considered separately). The Eq (4) addresses the overall social welfare impact of OOP heath care payments as a population share weighted average of the subgroups’ social welfare impact levels. This requirement has proved to be of great use in empirical analyses of regions, ethnic groups and other subgroups defined in a variety of ways. In this case, the overall welfare impact falls if social welfare decreases in one subgroup and is unchanged in other subgroups, given that the subgroup populations are fixed. On the other hand, the level of welfare impact in a given subgroup may be lower (or higher) than the overall welfare impact, and this has a direct effect on the overall welfare level. If we multiply the level of welfare impact of a given subgroup by the population share of that subgroup, *j*, that is *θ*_*jF*_, this can be viewed as the contribution of a subgroup to the overall welfare impact. All the subgroups’ contribution must then add up to one. This decomposable welfare impact measure avoids encountering the situation in which each effort to improve local welfare succeeds in increasing welfare, yet the measure of total welfare impact falls. It also avoids the residual component in some traditional decomposition analyses by using traditional socioeconomic health inequality indices, such as the concentration index which is not additively decomposable [10,26,38,39].

#### The marginal dominance welfare approach

In order to verify the robustness of the welfare impact measure we move from a complete to a partial welfare ordering, in which we are now interested in making comparisons between two distributions or alternatives. In this approach, we gain from having robust results to those obtained in Eq (4), but we lose in being able to provide a ranking, as not all the alternatives of interest may be ordered. For this, we extend the analysis proposed by Garcia-Diaz and Sosa-Rubi [20] incorporating the concept of marginal stochastic dominance curves to also evaluate the effect of OOP health care payments on social welfare (see S1 Text). Following the definition in (2), the resulting marginal dominance curves are the OOP health payment dominance curve of order *s*, *HD*^*s*^ (see supporting information S2 Text).

The OOP health payment dominance curve of order *s* for subgroup *j*, *HD*^*s*^_*j*_, can be interpreted as the weighted cost in terms of OOP health payments incurred by subgroup *j*. The order *s* refers to the normative (or ethical) judgments involved, in which it is possible to vary attitudes to inequality as it is measure with *ε* in the social welfare measure proposed in Eq 2. This attitude to inequality parameter is related to the concern for equity parameter defined in Eq (2), therefore the results are parallel, with the difference that the *HD*^*s*^_*j*,_ refers to a curve, while Eqs (2) and (4) are indexes. The *HD*^*1*^_*j*_ curve is the density of OOP health payments paid by households in subgroup *j*, while the *HD*^*2*^_*j*_ curve represents the cumulative OOP health payments paid by households in subgroup *j*. These results show that by relaxing assumptions for the case of *s = 1*, one can, a priori, rank households as relatively more deserving (high weight *β*^*h*^) or a relatively less deserving (low weight *β*^*h*^), whilst in the case of *HD*^*1*^_*j*_ curve, it just ranks the amount of the OOP health payments without any concern for equity. Although we can say that *HD*^*2*^_*j*_ is similar to a concentration curve, the difference between *HD*^*2*^_*j*_ and a concentration curve is that *HD*^*2*^_*j*_ accumulates over incomes while the concentration curves accumulates over population percentiles. The *HD*^*s*^_*j*_ curve also allows us to test for high-order OOP health impact dominance curves, putting greater weight on OOP health payments of those households in the lower part of the income distribution, i.e., the poorest households.

## Analysis and results

Table 1 shows the population’s main characteristics before and after propensity score matching. Before the matching, most of the differences of descriptive variables that compare SPS-insured and uninsured households are statistically significant: it is possible to identify that households with SPS tend to have lower education levels, a lower socioeconomic condition, are mainly located in rural areas from Southern Mexico, and have greater SPS penetration index. These differences disappear after matching (see columns 3 and 4).

Table 2 presents mean consumption at every decile and OOP health care payments for SPS beneficiaries and the uninsured population. As we would expect mean consumption ratio increases as the income decile grows (column 1). Counter-intuitively, overall the cumulative proportion of OOP health payments among SPS beneficiaries is larger at every decile level and higher than that of uninsured households (columns 3 and 5), apparently showing that SPS-insured households are spending more than their uninsured counterparts. Similar results were found in Laurell (2015)[40] who, when comparing average quarterly out-of-pocket expenditure by deciles, observed that SPS beneficiaries show greater out-of-pocket expenditures compared to those who are uninsured at lower decile levels. However, this result at different decile levels may not display the overall performance of the program on household financial protection according to income distribution and its related household weights. Other studies have also remarked on this descriptive approach for analyzing the SPS income distribution impact, showing inconclusive results regarding the comparison of differences in income distribution after OOP health payments between SPS-insured and uninsured households [13,16].

**Table 2:** Decile expenditure ratio and cumulative out-of-pocket (OOP) health payments 2010.

Decile	Mean consumption ratio	With Seguro Popular de Salud		Without any health insurance	
		Proportion of OPP health payments	Cumulative proportion OPP health payments	Proportion of OPP health payments	Cumulative proportion OPP health payments
	(1)	(2)	(3)	(4)	(5)
1^st^	1.00	0.03	0.03	0.01	0.01
2^nd^	1.80	0.06	0.09	0.03	0.04
3^th^	2.46	0.07	0.16	0.04	0.08
4^th^	3.14	0.07	0.23	0.04	0.12
5^th^	3.93	0.09	0.32	0.05	0.17
6^th^	4.89	0.08	0.40	0.07	0.24
7^th^	6.12	0.10	0.50	0.07	0.30
8^th^	7.91	0.15	0.65	0.12	0.42
9^th^	10.9	0.16	0.80	0.11	0.53
10^th^	24.4	0.20	1.00	0.48	1.00

**Note:** Estimations among matching sample. Matching process was performed using all variables in Table 1 and using single nearest neighborhood algorithm including: caliper = 0.001, non-replacement and common support.

Table 3 presents the distributional approach result for different levels of inequality aversion, ε parameter (note that as ε is higher, a larger weight is provided to the poorer household when the impact of OOP health payments on income distribution is measured). Panel A analyzes different population subgroups taking into account the household composition among SPS-insured and uninsured households. When we take the population as a whole, the beneficiaries of SPS have a lower proportion of OOP health payments (42.95%) than households without insurance (57.05%) (ε = 1, see column 1). As we take higher values of ε, the welfare weights are raised to the power of 2 and 3, and the distributional welfare impact in favor of SPS insured households is higher than the one shown by uninsured households. In other words, poorer SPS-insured households tend to have lower OOP health payments than their uninsured counterparts: the proportion of OOP health payments among SPS-insured and uninsured households is 19.16% and 80.84% respectively for ε = 2 and 0.55% and 99.45% respectively for ε = 3. In the analysis of different population subgroups among SPS-insured and uninsured households, SPS impacts positively, in terms of OOP health payments, the welfare of households with people over 65 years old and no children. In this subgroup, SPS-insured households present a lower OOP health payments share compared to those of uninsured households (5.36% vs. 19.89%, respectively). As we assign higher weights to poorer households, this trend is remarkably evident: for ε = 2 the share of OOP health payments is 3.57% for SPS-insured households vs 64.24% for uninsured households. For ε = 3, the share of OOP health payments is 0.16% for SPS-insured households vs 99.45% for uninsured households. On the other hand, we found that SPS-insured households with children and SPS-insured households with children and older adults, tend to have greater OOP health payments shares compared to uninsured households along all epsilon (ε values).

**Table 3:** Distributional welfare impact of OOP health payments among insured and uninsured groups of population.

	Level of inequality aversion					
	ε = 1		ε = 2		ε = 3	
	Health Payments	% share	Health Payments	% share	Health Payments	% share
	(1)	(2)	(3)	(4)	(5)	(6)
**PANEL A. DEMOGRAPHIC CHARACTERISTICS**						
Households with Seguro Popular de Salud						
With children and older adults^a^	295.04	0.45%	0.016	0.34%	2.078E-06	0.02%
With children and no older adults^b^	16,309.64	25.12%	0.508	10.73%	2.571E-05	0.25%
With older adults and no children^c^	3,482.94	5.36%	0.169	3.57%	1.708E-05	0.16%
Without children and without older adults^d^	7,798.33	12.01%	0.215	4.53%	1.242E-05	0.12%
Overall	27,885.95	42.95%	0.908	19.16%	5.729E-05	0.55%
Households without any health insurance						
With children and older adults^a^	135.96	0.21%	0.004	0.09%	1.664E-07	0.00%
With children and no older adults^b^	8,888.61	13.69%	0.231	4.88%	1.177E-05	0.11%
With older adults and no children^c^	12,917.40	19.89%	3.044	64.24%	1.022E-02	97.50%
Without children and without older adults^d^	15,101.78	23.26%	0.551	11.62%	1.933E-04	1.84%
Overall	37,043.74	57.05%	3.830	80.84%	1.043E-02	99.45%
**PANEL B. GEOGRAPHIC LOCATION**						
Households with Seguro Popular de Salud						
Large cities^e^	4,925.31	7.26%	0.148	1.96%	7.939E-06	0.09%
Medium cities^f^	1,614.54	2.38%	0.047	0.61%	1.892E-06	0.02%
Small cities^g^	3,122.88	4.60%	0.086	1.14%	3.467E-06	0.04%
Rural areas^h^	16,528.48	24.35%	0.730	9.63%	7.359E-05	0.79%
Overall	26,191.21	38.59%	1.011	13.34%	8.688E-05	0.93%
Households without any health insurance						
Large cities^e^	31,953.43	47.07%	1.117	14.74%	5.852E-05	0.63%
Medium cities^f^	1,975.60	2.91%	0.109	1.44%	1.282E-03	13.73%
Small cities^g^	2,665.88	3.93%	3.312	43.70%	3.049E-04	3.27%
Rural areas^h^	5,092.80	7.50%	2.030	26.78%	7.603E-03	81.44%
Overall	41,687.70	61.41%	6.568	86.66%	9.248E-03	99.07%

**Note:** Estimations made with the Income and Expenditure National Survey 2010 from Mexico.

^a^Children less than 11 yrs. old and adults greater than 65 yrs. old.

^b^Children less than 11 yrs. old and no adults greater than 65 yrs. old.

^c^Adults greater than 65 yrs. old and no children less than 11 yrs. old.

^d^Children less than 11 yrs. old and without adults greater than 65 yrs. old.

^e^Cities with more than 100,000 inhab.

^f^Cities between 15,000 and 99,999 inhab.

^g^Cities between 2,500 and 14,999 inhab.

^h^Cities with less than 2,500 inhab.

Matching process was performed using all variables in Table 1 and using single nearest neighborhood algorithm including: caliper = 0.001, non-replacement and common support.

Table 3 (Panel B) displays the distributional impact for insured and uninsured households in different geographical locations. Households affiliated to SPS and living in larger cities have more welfare protection against OOP health payments in comparison to those without insurance living in the same areas. The proportion of the OOP health payments is 7.26% among SPS-insured households compared to 47.07% among uninsured households. In contrast, SPS-insured households living in small cities and rural areas spend more than their counterparts (see column 2). As long as we provide higher weights to poor households (for ε = 2 and ε = 3), the share of OOP health payments moves towards uninsured households living in small cities and/or living in rural areas (see columns 4 and 6). This means that insured poorer households in small or rural areas tend to have a lower proportion of OOP health payments than uninsured poorer households.

**Fig 1:**
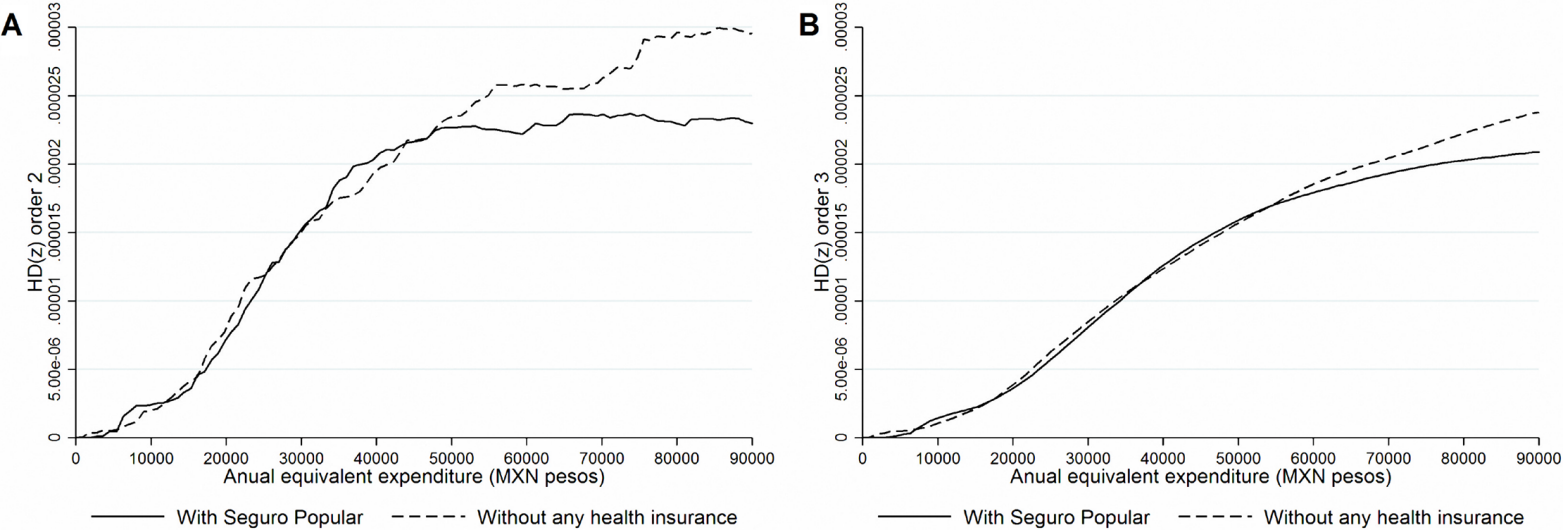
Health payments dominance curves for all population. **A.** Second degree comparison. **B.** Third degree comparison. **Note:** Estimations made with the Income and Expenditure National Survey 2010 from Mexico. Matching process was performed using all variables in Table 1 and using single nearest neighborhood algorithm including: caliper = 0.001, non-replacement and common support.

We reported a simple graphical method to establish the robustness of the numerical distribution comparisons that were shown in Table 3. Instead of obtaining cardinal results, such as those in Table 3, we use pair-wise marginal dominance (HD, distributional curves) comparisons between different subgroups. Fig 1 presents the OOP health payment dominance curve HD for order 2 (note that curves with higher order or higher degree assign larger weight to distributional changes on the poorest households’ incomes) comparing the SPS household beneficiaries and households without insurance. In Fig 1A, we found no conclusive results for second degree marginal stochastic dominance, as the curve of the SPS beneficiaries crosses with that of the uninsured at several points along income distribution. At the third degree of marginal stochastic dominance (Fig 1B), we still found no conclusive results since there are crossings of the marginal dominance curves at the very bottom of the distribution.

**Fig 2:**
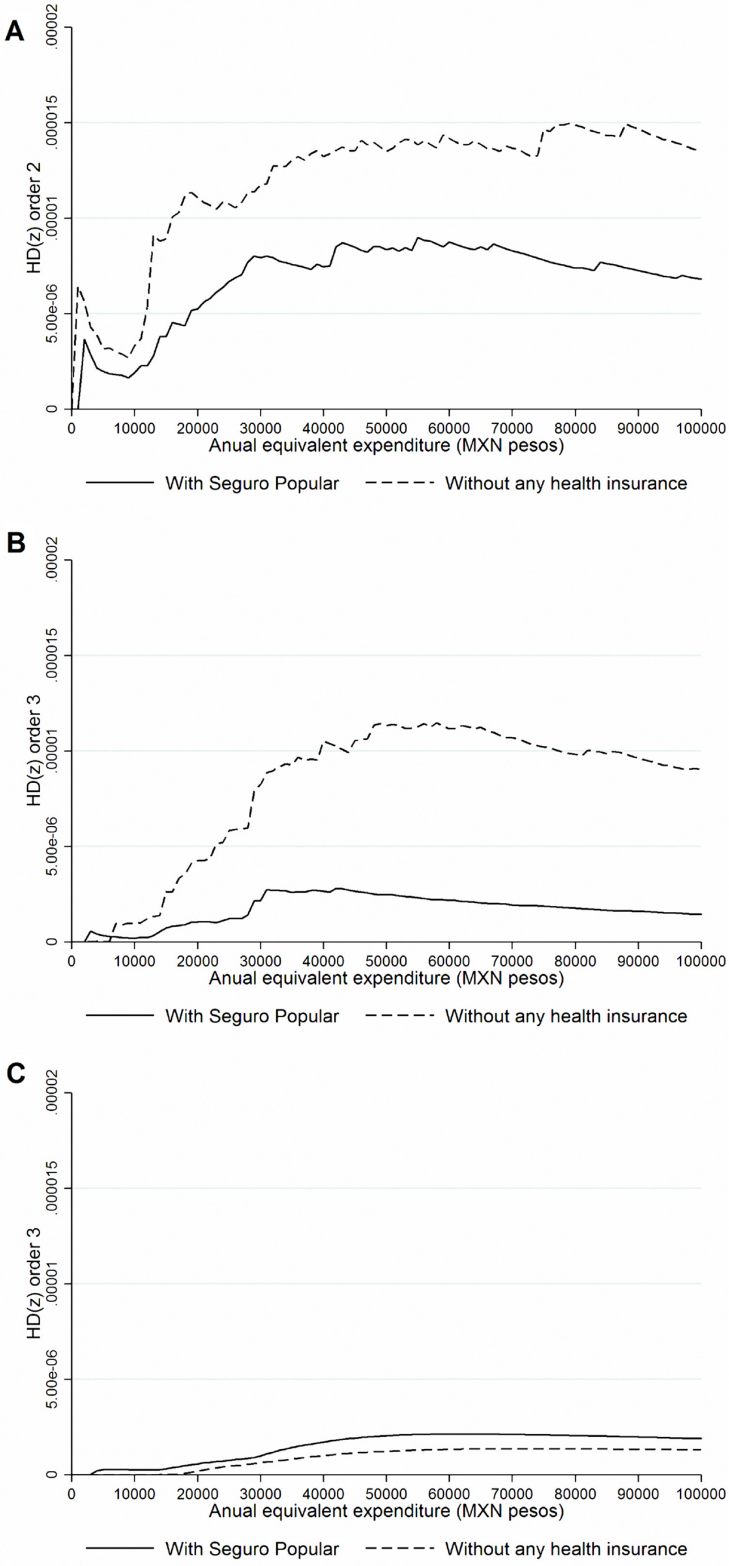
Health payments dominance curves according to socio-demographic characteristics. **A.** Households with older adults and without children. **B.** Households with children and without older adults. **C.** Households with older adults and with children. **Note:** Expressed in US$ and per women 15–49 years of age (at constant prices of 2011). **Includes women without any type of health insurance and those who reported being affiliated to the Seguro Popular.

When we focus on specific subgroups of population we found robust results. In Fig 2A and 2B, we compare second-degree health care payments marginal dominance curves for households with older adults without children and households with children and without older adults. We confirm that those SPS-insured households with older adults and no children have a lower share of OOP health care payments than their uninsured counterparts. On the other hand, we found no marginal dominance for households with children and without older adults; this is due to crossings in the lower part of the distribution. If we consider households with older adults and with children, we find OOP third-degree health care payment marginal dominance in favor of uninsured households (see Fig 2C).

**Fig 3:**
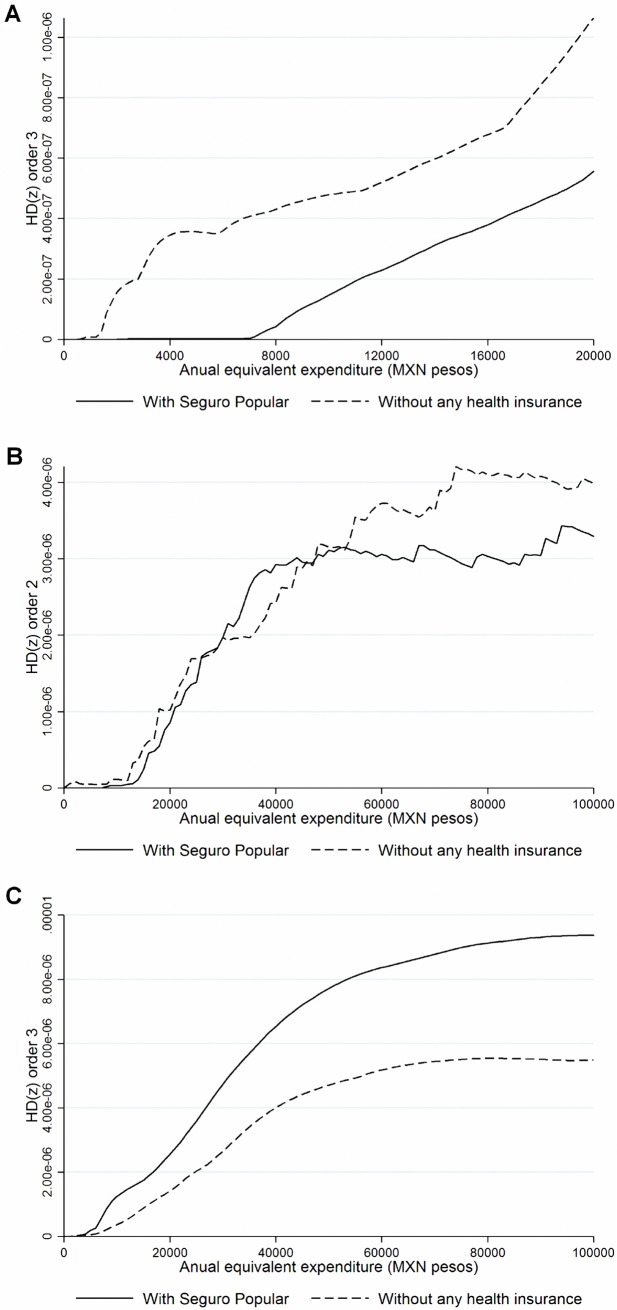
Health payments dominance curves according to household’s geographic location. **A.** Households living in large cities^a^. **B.** Households living in small cities^b^. **C.** Households living in rural cities^c^. **Note:** Estimations made with the Income and Expenditure National Survey 2010 from Mexico. ^a^Cities with more than 100,000 inhab. ^b^Cities between 2,500 and 14,999 inhab. ^c^Cities with less than 2,500 inhab. Matching process was performed using all variables in Table 1 and using single nearest neighborhood algorithm including: caliper = 0.001, non-replacement and common support.

Fig 3 displays results of the effect of the SPS program by geographical location. We found third-degree marginal dominance in larger cities (Fig 3A) and rural areas (Fig 3C), confirming our findings using the indexes shown in Table 3. The marginal dominance result favors SPS beneficiaries in larger cities when compared with uninsured households in larger cities. In contrast, in rural areas SPS-insured households tend to spend more than insured households. For small-sized cities (Fig 3B), there are no conclusive results once marginal dominance methods are applied and the same was found for medium-sized cities (results not shown).

## Conclusions and discussion

We use distributional welfare impact and the marginal welfare dominance approach to analyze the effect of *Seguro Popular de Salud* (SPS) on households’ welfare after OOP health payments, in different population groups in Mexico. The methodology shares some similarities with the concentration index, yet it allows us to decompose the welfare impacts in terms of OOP health care payments into its sources by analyzing its subgroup components.

Overall, our analysis suggests there is a positive welfare impact of SPS at different levels of inequality aversion parameters for insured households compared to uninsured households. However, these results are non-conclusive according to the verification of the robustness of the numerical approach with the marginal dominance curves. As Laurell (2015)[40] pointed out, OOP health expenditures are related to specific demographic characteristics of the population. Accordingly, we found robust results in favor of the effect of SPS on households’ welfare when examining specific subgroups of the population: household beneficiaries of SPS with older adults and no children have a lower proportion of OOP health care payments than the uninsured counterparts, which shows the financial protection that SPS is offering to households with older adults. This is an important achievement of the SPS program given the substantial vulnerability of households with older adults, insomuch as they are more vulnerable to suffer chronic diseases that could represent a financial burden for poor households and hence face catastrophic spending [41–47]. This is consistent with SPS’ main objectives, particularly regarding the aim of offering financial protection to households with greater financial vulnerability. We complement previous findings that have shown a positive effect of SPS on access to health care [48], health resources such as insulin [49], and improved access to health services to prevent chronic and infectious diseases [50] among older adults, with robust results regarding SPS’ welfare protection of older adults’ households.

The size of cities where households live also appears to play an important role when analyzing the scope of SPS on households’ welfare among different regions. We found robust results indicating that households who live in larger cities and are beneficiaries of SPS have a smaller share of OOP health care payments than those that are uninsured living in the same areas. Although results are not robust regarding the impact of SPS on the welfare of insured households living in smaller cities or rural areas with respect to uninsured households, as long as we provided higher weight to poor households from small cities or rural areas to estimate the welfare index proposed in this paper, the share of the OOP health payments was smaller among SPS-insured households compared to uninsured households living in these areas. These inconclusive results could be related to the fact that both SPS-insured and uninsured households located in small cities and rural areas continue to have a high share of their income allocated to OOP health payments. This could be consistent with a recent evaluation of the SPS showing that this program has reduced catastrophic spending among households living near larger medical units, located in large cities, but not for poorer households living in small locations who still have the largest OOP health care payments [51]. In line with this, recent evidence has shown that access to health care in rural communities among SPS-insured households is much lower than access among insured households in urban localities [51], with particularly pronounced gaps among indigenous groups, low socioeconomic status and geographically isolated populations. This might mean that insurance coverage alone may not protect households against catastrophic health expenditures, but also requires the improvement of access to health resources and services, as is case of urban areas. This calls to overcome the structural failures that the Mexican public health system faces to ensure effective access to health services and resources to populations living in small cities or rural areas. The implementation of SPS intended to strengthen health resources availability during its execution. However this has not been enough to ameliorate the effective access to health resources to the eligible population.

Grogger J. et al. [51] highlighted the importance of the role of access to health services in smaller and rural areas in order to enhance the scope of SPS. Although SPS intended to ensure a minimum infrastructure and resources required to provide the essential medical interventions included in its insurance coverage at the state level [46], these efforts have not been enough to have a positive impact on the reduction of the share of OOP health payments that households living in rural areas or small towns face. It seems that those households prefer or are forced to consume private services. This structural failure in the system, in which SPS-insured households did not find effective care through the public system network causes considerable limitations in the SPS achievements concerning household’s welfare protection [52].

The results of this study have some limitations that should be noted. First, we used a self-reported measure of outcome variables, SPS exposure and covariables, while data were not available on length of program exposure. Second, health expenditure is largely influenced by health status and the 2010 ENIGH provides partial information on diagnoses or any other clinical information for household members. In the models that correct for self-selection bias, we constructed dummy variables that indicate whether or not any member of the household had any kind of health ‘necessity’ in the last 12 months. However, we still have problems with the magnitude of the effect of the health ‘necessity’ on OOP health payments given that, in the ENIGH, we were not able to identify the type of health problems that each individual faced. Third, this study was based on a household survey that asked individual members about their spending, which is related to recall bias. However, the survey is considered “gold standard” when analyzing household income and expenditure in Mexico because it is the most detailed national representative survey available. Fourth, this analysis is subject to the limitations of all observational studies, such as potential omitted variables bias. However, we used a rigorous matching method to assess the robustness of our results and reduced a potential bias (in observable variables) in the selection of participation of SPS individuals.

Despite the limitations, the results obtained on the distributional effect of SPS were robust when analyzing different population subgroups. Our results suggest that access to SPS ensures household welfare protection against OOP health payments, particularly among vulnerable households, such as those with older adults. Additionally, we found a positive distributional effect of the SPS only in large urban cities, which suggests that SPS-insured and uninsured households located in rural areas and small cities required better access to medical resources to avoid greater household health expenditures.

These results provide evidence that emphasizes the importance of the design of strategies that ensure not only access to insurance coverage for the poor, but also their access to health resources and health care, in order to effectively reduce the financial burden from OOP health expenditures.

## Supporting information

S1 Table Predictors of participation in *Seguro Popular de Salud* (SPS). **Note:** Matching process was performed using single nearest neighborhood algorithm including: caliper = 0.001, non-replacement and common support. ^Φ^At municipality level. ^φ^Proxy of household socioeconomic level.
(DOCX)

S1 Fig Bias reduction and common support after propensity scores matching. **A.** Standardized bias (%)
**B.** Propensity score histogram
**Note:** Matching process was performed using all variables in Table 1 and using single nearest neighborhood algorithm including: caliper = 0.001, non-replacement and common support. HH: Head of household.
(TIF)

S1 Text OOP health payments dominance curves. (DOCX)

S2 Text Decomposing distributional welfare impacts into subgroups. (DOCX)
