# Supplementary material for: Welfare effects of health insurance in Mexico: The case of Seguro Popular de Salud
Source: PLoS One. 2018 Jul 2;13(7):e0199876. doi: 10.1371/journal.pone.0199876 (PMC6028097; doi:10.1371/journal.pone.0199876)
Supplement: S1 Table — Note: Matching process was performed using single nearest neighborhood algorithm including: caliper = 0.001, non-replacement and common support. ΦAt municipality level. φProxy of household socioeconomic level. (DOCX) [file pone.0199876.s001.docx]

**S1 Table.** Predictors of participation in *Seguro Popular de Salud* (SPS)

|  | **Coeff.** | **Std. Err.** | **z** | **P>\|z\|** |
| --- | --- | --- | --- | --- |
| Head of household |  |  |  |  |
| Male | -0.41 | 0.06 | -7.12 | 0.00 |
| Age (Ref.: <20 yrs.) |  |  |  |  |
| 20-39 yrs. | -0.07 | 0.26 | -0.28 | 0.78 |
| 40-59 yrs. | 0.62 | 0.05 | 11.4 | 0.00 |
| 60-79 yrs. | 0.57 | 0.05 | 11.7 | 0.00 |
| Marital status: married | 0.77 | 0.06 | 13.9 | 0.00 |
| Schooling (Ref.: ≥13 yrs.) |  |  |  |  |
| Nothing (0 yrs.) |  |  |  |  |
| Primary (1-6 yrs) | -0.06 | 0.06 | -0.99 | 0.32 |
| Secondary (7-9 yrs.) | -0.11 | 0.07 | -1.52 | 0.13 |
| High school (≥10 yrs.) | -0.85 | 0.08 | -11.1 | 0.00 |
| Asset Index^φ^ | -0.09 | 0.01 | -6.30 | 0.00 |
| Members with any health problem (%) | 0.37 | 0.06 | 6.29 | 0.00 |
| Residency area (Ref.: Rural) |  |  |  |  |
| Urban | -0.09 | 0.05 | -1.69 | 0.09 |
| Metropolitan | -0.31 | 0.06 | -4.88 | 0.00 |
| Geographical area of residency (Ref: South) |  |  |  |  |
| North | 0.30 | 0.05 | 5.83 | 0.00 |
| Center | 0.08 | 0.04 | 1.74 | 0.08 |
| Penetration of Seguro Popular de Salud^Φ^ | 2.52 | 0.12 | 20.4 | 0.00 |
| Intercept | -1.49 | 0.11 | -13.6 | 0.00 |
| Observations |  |  |  | 14,731 |
| Prob > χ^2^ |  |  |  | 0.00 |
| Pseudo R^2^ |  |  |  | 0.15 |

Note: Matching process was performed using single nearest neighborhood algorithm including: caliper=0.001, non-replacement and common support. ^Φ^At municipality level. ^φ^Proxy of household socioeconomic level.
