# Supplementary figures and images for: Welfare effects of health insurance in Mexico: The case of Seguro Popular de Salud
Source: PLoS One. 2018 Jul 2;13(7):e0199876. doi: 10.1371/journal.pone.0199876 (PMC6028097; doi:10.1371/journal.pone.0199876)

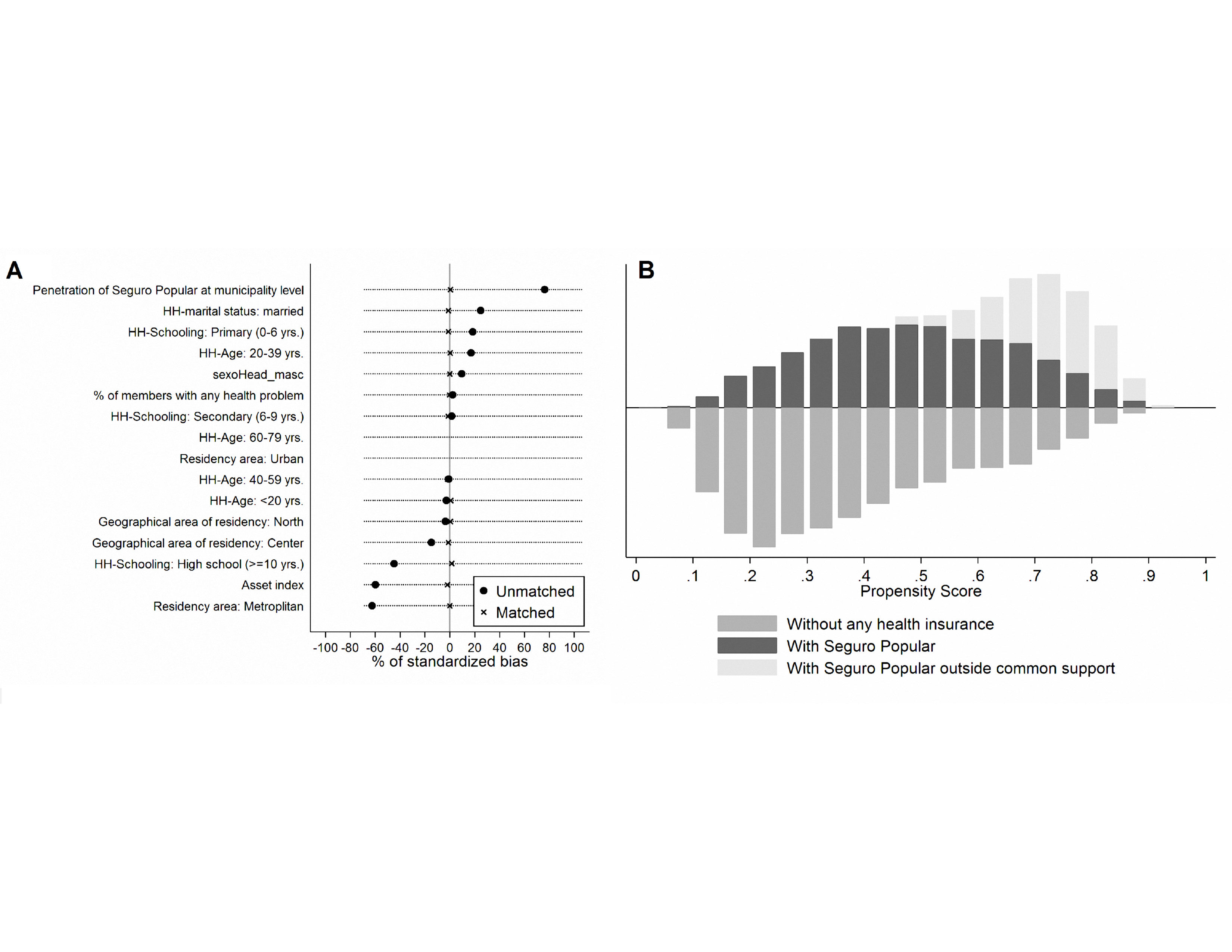

Supplement: S1 Fig — A. Standardized bias (%) B. Propensity score histogram Note: Matching process was performed using all variables in Table 1 and using single nearest neighborhood algorithm including: caliper = 0.001, non-replacement and common support. HH: Head of household. (TIF) [file pone.0199876.s002.tif]
