# Supplementary material for: Welfare effects of health insurance in Mexico: The case of Seguro Popular de Salud
Source: PLoS One. 2018 Jul 2;13(7):e0199876. doi: 10.1371/journal.pone.0199876 (PMC6028097; doi:10.1371/journal.pone.0199876)
Supplement: S1 Text — (DOCX) [file pone.0199876.s003.docx]

**S1 Text.** OOP health payments dominance curves

The analysis is based on previous results and applications of the marginal stochastic dominance analysis. We analyze welfare functions that belong to a class of order *s* (where *s*=1,2,3,…), *U^s^*. The order *s* refers to the normative (or ethical) judgments involved, then, a normative order *s* characterizes the class also of order *s*. For that purpose, we require that the function *u*(*y^i^,p*) be a continuous function and *s* -time differentiable with respect to its first argument over [0,*a*], which we represent as, *u^s^*(*y^i^,p*). Therefore, the normative interpretations of the differences classes characterized involve different appreciations of how the welfare function is affected by changes in income. For instance, first order classes of welfare functions, when *s=*1, group all those indices that show a social improvement when the income of some household increases while no other income changes. Second order classes of welfare functions, when *s=*2, show a social improvement when a mean-preserving distributive transfer from a richer to a poorer household occurs, which correspond to the Pigou-Dalton principle on social judgment. As the order *s* increases, the welfare function becomes more sensitive to the effect of income changes occurring at the bottom of the distribution, in the poorest households. We now define the stochastic dominance curve associated with a given distribution of income:

$\text{D}^{\text{s}}\text{=}\frac{\text{1}}{\left( \text{s-1} \right)\text{!}}\int_{\text{0}}^{\text{a}} \left( \text{a-y} \right)^{\text{s-1}}\text{dF(y)}$ (1)

where it is said that distribution describing situation *j* dominates (stochastically) distribution *k* at order *s*, if *D^s^_k_*(*y*)≥*D^s^_j_*(*y*) for all incomes in the distribution. In our analysis we are interested not in comparisons of two income distributions, but rather in the marginal effect of a given income distribution as a result of the OOP health care payments of households. Therefore, we follow Garcia-Diaz and Sosa-Rubi [19] and consider marginal stochastic dominance curves that result from the effect of OOP health payments in a given income distribution: the OOP health payments dominance curve of order *s*, *HD^s^*, defined as,

$\text{HD}^{\text{s}}\text{=}\frac{\text{∂}\text{D}^{\text{s}}\text{(y)}}{\text{∂h}}\text{=}\left\{ \begin{aligned} \text{∆h}\left( \text{x} \right)\text{f(x)} \\ \frac{\text{1}}{\left( \text{s-2} \right)\text{!}}\int_{\text{0}}^{\text{a}} \left( \text{x-y} \right)^{\text{s-2}}\text{∆h}\left( \text{x} \right)\text{dF(x)} \end{aligned} \right.$ (2)

The curve in (2) allow us to test a necessary and sufficient condition for distribution *j* to be *s* -order welfare dominant, with respect to distribution. *k*, that is, to reduce less social welfare weakly, for all *W ϵ U^s^* and for a given s *ϵ* {1,2,3,…} such that,

*HD^s^_j_*(*y*)-H*D^s^_k_*(*y*)≤0 (3)
