# Supplementary material for: Welfare effects of health insurance in Mexico: The case of Seguro Popular de Salud
Source: PLoS One. 2018 Jul 2;13(7):e0199876. doi: 10.1371/journal.pone.0199876 (PMC6028097; doi:10.1371/journal.pone.0199876)
Supplement: S2 Text — (DOCX) [file pone.0199876.s004.docx]

**S2 Text.** Decomposing distributional welfare impacts into subgroups

We start by defining the social welfare impact of OOP health care payments made by a household *i* as:

$\text{∆W=}\int_{0}^{\text{a}} \text{β}^{\text{i}}\text{∆}\text{h}^{\text{i}}\text{f}\left( \text{y} \right)\text{dy}$ (1)

where, $y$ is the income of household *i*, *Δh^i^* is the change in the amount of out-of-pocket health care payments paid by household *i*, *f(y)* is the density distribution of income defined over [0,a], where *a* is the maximum level in the income space. *β^i^* is the social valuation of extra-income to household *i*, that is the social weight according in terms of income a given household has in a society.

For the decomposition, we let *F_j_(y)* be the distribution function for the income *y* of households in the *j*th subgroup (where *j*=1,2,…,*k*), and *θ_jF_* be subgroup’s *j* population share (the number of household in subgroup *j* divided by the total number of households). If *a* is the income level exceeding the maximum income in any subgroup, then we have *F_j_(a)=1*, for all *i*, and . The social welfare impact in (1) for the population can be re-expressed as the average of subgroup welfare impacts, as follows:

$\text{∆W=}\sum\text{θ}_{\text{jF}}\int_{0}^{\text{a}} \text{β}^{\text{i}}\text{∆}\text{h}^{\text{i}}\text{f}\left( \text{y} \right)\text{dy}$ (2)

Here the contribution of subgroup $j$to total welfare impact is found by weighting the subgroup welfare impact value by its population share and then expressing this as a percentage of total welfare impact. For a decomposable measure this sum to 100%; for no-decomposable poverty measure this sum may exceed 100%.
